# Rent increase strategies and distributive justice: the socio-spatial effects of rent control policy in Amsterdam

**DOI:** 10.1007/s10901-017-9573-2

**Published:** 2017-10-24

**Authors:** Arend Jonkman, Leonie Janssen-Jansen, Frans Schilder

**Affiliations:** 1 Department of Human Geography, Planning and International Development, Amsterdam Institute for Social Science Research, Urban Planning, University of Amsterdam, Nieuwe Achtergracht 166, 1018 WV Amsterdam, The Netherlands; 2 Environmental Sciences, Land Use Planning Group, Wageningen University, Droevendaalsesteeg 3, Building 101, 6708 PB Wageningen, The Netherlands; 3 PBL Netherlands Environmental Assessment Agency, Bezuidenhoutseweg 30, 2594 AV The Hague, The Netherlands

**Keywords:** Housing policy, Affordability, Distributive justice, Rent price strategy, Social housing

## Abstract

Rent controls and rent setting regulation in different contexts incorporate and balance different aims, in particular when securing affordability and the effective distribution of scarce housing by incorporating market mechanisms. As rent policy is frequently discussed in terms of affordability or market functioning in broad terms, small-scale distributive socio-spatial effects are often not regarded. In this paper, three strategies under the new rent sum policy are compared against the former policy and practice for Amsterdam, the Netherlands, to observe the effects of distributive justice. The new rent policy partly decentralizes rent increase decisions from the national level to local authorities and housing associations. Using microdata on all social housing units and their tenants’ distributive justice, outcomes under the former policy and practice are observed for a 6-year period (2008–2014) and the effects of three different rent increase strategies under the new rent sum policy are forecasted for the same period, combining an ex ante and an ex post evaluation. The possibilities for housing associations to vary rent increases for different groups of tenants in order to improve distributive justice outcomes are explored. Results show that all three possible strategies decrease the observed affordability gap between new and long-term tenants. Valuing the distributions of these strategies by applying two different standards for distributive justice shows the rent sum policy may only result in modest improvements.

## Introduction

Housing affordability has become a central issue on urban agendas around the world due to sky-rocketing house prices and rents, providing profits and opportunities for few while imposing losses and threats to others. Various local and national governments have developed policy measures to secure housing affordability for low- and middle-income households. Policies can focus on the aspects of the supply of affordable housing (e.g., US tax credits incentivizing developers to include affordable housing in new developments; Schwartz 2010), the demand for affordable housing (e.g., different forms of housing allowance or housing vouchers; Priemus et al. 2005), or improve the efficient use of available means (e.g., UK policy referred to as the ‘bedroom tax’ which seeks a more efficient use of the current scarce public housing stock; Gibb 2015). All these policies aim to change ‘who gets what’ of scarce resources and thus shapes the distributive justice. ‘Distributive justice involves a moral judgement of an allotment of receipts among recipient units’ (Cohen 1987, p. 24). Receipts can have different forms, like goods, services, opportunities, or even duties. The moral judgement can be based on different standards (e.g., equality, merit, benevolence, priority), which also depend on the nature of the good distributed (Frankena 1966). With respect to social housing, different qualities of housing are distributed among different households. Another example consists of rent policies that have an impact on both the supply of affordable housing and the efficient use of available means. The supply is affected by limiting rents and rent increases, while the efficient use of available means is adjusted by aligning rents and household needs.

Various policies have been introduced in the Netherlands, especially since the start of the economic crisis in 2008, to primarily improve the effective use of the existing social housing stock. Recently introduced policies include, for example, altering the extent to which maximum allowed social housing rents are connected to local real estate values and changing the target group definition of households eligible for social housing. The recently (in 2017) introduced policy *rent sum policy* decentralizes national rent setting and assigns the responsibility to local governments and housing associations. Consequently, local governments and housing associations gain explicit freedom to determine how to distribute rent increases across their tenants as long as the sum of all increases does not exceed last year’s inflation + 1%. Furthermore, housing associations and municipalities are allowed to jointly install additional criteria for rent increases and are granted significant opportunities to strategize on future rent increases. This provides—at least in theory—the potential to adjust the strategy based on socio-spatial specificities in a city and needs of households. Vulnerable household groups or deprived neighborhoods could receive a lower rent increase for a comparable unit than those better off, eventually changing the distribution of affordability and thus the distributive justice. Local governments could develop several implementation strategies with potentially different results.

The aim of this paper is to compare—for the city of Amsterdam, the Netherlands—three strategies under the new rent sum policy in terms of the distribution of affordability and evaluate this in terms of distributive justice outcomes. By forecasting the effects for the 2008–2014 period, the distributive outcomes of three different rent increase strategies of implementing the rent sum policy will be compared to the development under the former policy and practice over the same period. Subsequently, it will be assessed whether housing associations are able to improve the distributive justice outcomes by adopting a specific rent increase strategy. The approach is innovative as it combines ex ante and ex post evaluations to assess the different policy implementation options. Furthermore, the analysis shows potential socio-spatial effects of each strategy at a detailed level. The research calculations use non-public household microdata from Statistics Netherlands (CBS) and housing unit microdata from the Federation of Amsterdam Social Housing Associations (AFWC) and Platform Woningcorporaties Noordvleugel Randstad (PWNR). The results not only shed light on the socio-spatial effects of the policies and strategies, but also provide relevant input for housing associations and municipalities in regard to policy implementation.

This paper continues with a discussion on rent control and price setting in Sect. 2. Section 3 addresses Dutch rent policy and rent setting practices. Sections 4 and 5, respectively, are about the relation between rental policy and distributive justice and the applied methods and data. In the last two sections, results are presented and conclusions are drawn.

## Rent control and price setting

Rents of housing rented out by for-profit landlords aiming to maximize earnings will follow current market rates. Social housing rents are usually determined differently. Kemeny (1981) described how rents can be lowered in comparison with market rates if a housing portfolio of a non-profit housing provider matures over time. Through rent pooling of older debt-free and newly financed units, rents can be averaged at below market rates. The total rent received should cover financial obligations, maintenance and management. New investments can be enabled through the maturation process in which mortgages are paid off over time improving the organizations balance. As a portfolio matures, the gap between pooled cost-rent and market rent increases. Making use of different types of rent regulation, rents in both the private rented and social rented sector are influenced. This paper focuses on social rented housing and how social rents are shaped by both rent controls and other rent setting regulation.

### Rent control

Rent controls have been introduced in many countries in times of housing shortage, especially in the wake of both world wars. Tight rent control, or even rent freezes, have been very popular, and strict controls have been in place in several countries like Spain, the UK, The Netherlands, Sweden and France (Deschermeier et al. 2016). A distinction can be made between early *first-generation* rent controls, that would freeze rents, and softer *second-generation* rent controls that aim to stabilize rents and limit yearly rent increases for current tenants (Arnott 1995; Dienstfrey 1981; Lind 2001). Both types of rent control exist in various forms, and all aim to improve housing affordability.

Though rent control is primarily related to government policy to secure affordability on the private rented sector (e.g., Haffner et al. 2008), rent control is applied to both private and social housing sectors. Most studies on the effects of rent control have, however, focused on the impact of rent controls on the earning potential of private landlords and on their decisions on maintenance (Arnott and Shevyakhova 2014; Lind 2015), investments and the sale of rental housing (e.g., Andrews et al. 2011; Autor et al. 2012; Block and Olsen 1981). Enström Öst et al. (2014) additionally show how rent control affects the socio-spatial distribution of housing. They discovered that implicit subsidies resulting from locational higher discounts on market rents in central areas of Stockholm are not effectively allocated to those with the highest need. Similar to what is found in Amsterdam (self-identifying reference), the flat distribution of rents can be regarded an inefficient implicit subsidy (Schilder and Conijn 2015) for those living in the most attractive parts of the city. Such implicit subsidies—consisting of the sum of the differences between current rents and market rents if rents would not have been regulated—may be part of both rent-controlled social and private rented sectors.

### Rent setting

Rent distributions—that would be very flat in case of rent pooling in a social housing sector—are affected by different policies and practices. Especially by relating rents to the household income, the housing cost, the housing quality or the housing market value, the way rents are set has been adjusted. Differentiation of rents based on these characteristics would hardly be reflected in a flat rent distribution: ‘comparatively flat rent structures that emerge under rent pooling give tenants little incentive to optimize their housing consumption’ (Walker and Marsh 2003, p. 2024). Besides, flat rent distributions may lead to exacerbated geographical differences in demand as housing decisions are not influenced through pricing (Walker and Marsh 2003). More popular areas may become even more wanted because of the greater implicit subsidy, especially resulting in longer waiting lists.

In the ideal–typical social rented sector, as described by Kemeny (1981), rents are evened out through rent pooling and related to the total cost of housing for the landlord (including construction, maintenance and management). Social housing is sometimes also referred to as cost renting (e.g., Kemeny 1981; Murie and Williams 2015). While some degree of rent pooling is allowed in many countries, Denmark is a rare example of a country in which social housing is required to be rented at cost rent of the individual dwelling. Consequently, older units that are often also more attractive have much lower rents than newer units (Vestergaard and Scanlon 2014).

Cost rent housing with a certain degree of rent pooling is often combined with setting rents based on the household income, the housing quality and/or the housing market value. In Hong Kong, for example, affordability was implemented as leading principle for rent setting in the public sector since the 1980s, making use of different rent-to-income ratios. Rent setting was based on the average affordability for tenants. The median household was supposed to have a rent-to-income ratio of at most 15% (Yip and Lau 2002). In Belgium and Ireland, rents are directly related to individual household incomes (Haffner et al. 2008). Incomes are also checked in these countries. In Belgium, also a base rent applies and the rent is partly determined by the value of the unit and the size of the family.

In the Swedish model, rents of new lettings are mostly similar to market rates, while inflation-following rent increases have resulted in increasing gaps between current rents and market rents for older dwellings (Svensson 1998). Recent reform, triggered by a state-aid complaint at the European Commission, forces municipal housing companies to act like market actors. Rent restructuring remains based on negotiations between tenant unions and housing providers, but now the private sector is included in the process. The first negotiations, however, did not show signs of major shifts toward rents of current tenants that better reflect demand (Elsinga and Lind 2013; Haffner et al. 2008).

In England, different rent setting policy reforms since the 1970s have aimed to further centralize rent setting (Marsh and Walker 2006). The 2000 Green Paper introduced a single formula for rent setting in which different aims are integrated. Four explicit objectives of the Green Paper were to make rents fairer and less confusing, reduce unjustifiable differences between local authority and housing association dwellings, improve the quality of management and make rents better reflect relevant qualities (Marsh and Walker 2006; Walker and Marsh 2003). Rent differences thus had to especially better reflect differences in quality and demand.

These examples show rent setting regulation can have different aims related to securing affordability and improving market functioning. While Swedish rent setting moves to market rents and Danish rent regulation is cost based, rent setting in Hong Kong (Yip and Lau 2002), Belgium and Ireland is primarily aimed at securing affordability (Haffner et al. 2008). The case of England shows a search toward centralization of rent setting, by applying—similar to Belgium—a single formula for rent setting (Tang 2008). In countries where rent setting is not primarily focused on securing affordability, this may increasingly depend on additional housing allowance schemes. In the Netherlands, the new rent sum policy sets rent setting and rent increase boundaries at the national level, but also explicitly provides more space to differentiate rent increase strategies. The boundaries for initial rent setting are based on the quality and market value of the dwelling, while the boundaries for rent increases also depend on the household income and the rent charged in comparison with the maximum allowed rent for the unit.

## Rent setting in Amsterdam

In this section, rent controls and rent setting practices in Amsterdam that were in effect in the 2008–2014 period are discussed, before describing the new rent sum policy that came into effect in 2017 in more detail. Along the way, important characteristics of social housing in the Netherlands and Amsterdam are described.

### Rent control and rent setting from 2008 until 2014

The social housing sector has become a dominant tenure in the twentieth century, especially in the first post-Second World War decades. While in many other contexts—including other Dutch cities—the social housing sector came in decline already in the 1980s, in Amsterdam the sector continued to grow until its peak in 1995. Currently, social housing in Amsterdam consists of about 45% of the total housing stock. While also a large part of privately owned rented housing is rent controlled,^1^ in this paper we focus on these housing association-owned regulated (i.e., rent controlled) housing units. After the government paid off all future subsidies owed to housing associations in 1995, the housing associations in the Netherlands became largely financially independent. Supply-side ‘brick-and-mortar’ subsidies were abolished and a revolving fund model to finance housing associations was introduced (Elsinga and Lind 2013). This revolving fund model allows for rent pooling and stipulates that financial obligations and investments (e.g., upkeep, new construction and investments in community development) are financed with revenues out of the organization’s housing stock and by acquiring capital on financial markets. While housing associations are no longer subsidized directly, they still have access to government-backed (i.e., cheaper) loans (Veenstra and van Ommeren 2015). Furthermore, housing associations do not have economic owners and therefore the demanded rate-of-return on equity is low. In specific cases, a negative rate-of-return can even be accepted in order to achieve certain social goals (Conijn 2011). The total cost of capital for housing associations is therefore significantly lower than for private investors.

In regards to rent control in the Netherlands, a distinction can be made between maximum allowed rent levels and maximum allowed rent increases. The maximum allowed rents for regulated housing are determined by an administrative valuation system based on points reflecting the quality of the unit. Units with a point-total of up to 142, corresponding to a maximum allowed rent (i.e., the liberalization level) of €699 in 2014, are rent controlled. Units with a higher point-total may—but do not have to—be rented out without the restriction of rent regulation policy. If the initial rent at the start of the tenancy is lower than the liberalization limit, the unit remains regulated for the whole tenancy. Since 2012, the point valuation system includes scarcity points. Additional points are allocated to dwellings that are located in popular areas with high average house prices.^2^

The regulation of yearly rent increases—the second part of the rent control—is for each year being determined by the government and was usually set at or around the rate of last year’s inflation. From 2013 onwards, however, rent increases for social housing have been made temporarily income dependent, using three different maximum rent increase levels for different income groups. In 2013, the maximum rent increase level for households with an income up to €33,614 was also set higher at inflation + 1.5% (see Table 1).

**Table 1:** Maximum allowed rent increases and percentage of maximum allowed rents in Amsterdam (*Sources*: AFWC 2008–2014; CBS 2017)

Year	Inflation previous year (%)	Max. allowed rent increase (%)	Average rent (€)	Share of max. allowed rent			
				Average	< 60%	60–90%	> 90%
2008	1.6	1.6	364	78	5	77	17
2009	2.5	2.5	377	78	6	75	19
2010	1.2	1.2	392	79	7	72	21
2011	1.3	1.3	401	80	6	70	25
2012	2.3	2.3	419	67^a^	28	68	4
2013	2.5	4^b^	439	68	27	65	7

^a^In 2011, scarcity points were introduced. Units located in areas with high housing values were allocated addition points, resulting in a higher maximum allowed rent for units in Amsterdam

^b^Rents of households with an annual income in excess of €33,614 could be increased with up to 4.5% and rents of households with an annual income in excess of €43,000 could be increased with up to 6%, after introduction of income-dependent rent increases

Housing associations, on average, have asked around 80% of the price they are legally allowed to charge for new tenants, while current tenants on average pay 72% of the maximum allowed rent (Aedes 2016a). Table 1 shows that the average social housing tenant in Amsterdam paid 78% of what was maximum allowed in 2008. After 2011, the share of maximum allowed rent that was on average charged dropped from 80 to 67%. This was the result of the introduction of scarcity points increasing maximum allowed rents for all social housing units in Amsterdam. On a national scale, scarcity points over time can result in a less flat rent distribution. Within the Amsterdam municipality, however, this effect is expected to be minimal, because all social housing units are allocated scarcity points. The gap between new and current tenants is the result of the inflation-following rent increases and rent harmonization—i.e., the rent hike if a unit is rented out to a new tenant.

The 2008 financial crisis and the subsequent policy changes have increased financial pressure on housing associations. Gruis and Van der Kuij (2012, p. 52) refer to the housing associations’ double crisis. The financial crisis resulted in stagnating sales, losses on new housing development projects due to revenue shortfalls and—for some housing associations—write-downs on interest swaps. The second crisis, that of policy changes, includes an extra levy imposed by the government on housing associations. This levy was introduced in 2013 and builds up in a few steps to an annual €1.7 billion in total. Furthermore, for-profit activities of housing associations have been restricted in 2011 and the social housing target group was redefined by introducing a maximum income level of €34,678 (level of 2014), also in 2011. The strong financial position has enabled housing associations to contribute to affordability. The increased financial pressure, however, also influences housing association rent setting and rent increase decisions as well as decisions on the sale of social housing units (Elsinga and Wassenberg 2014).

### The new rent sum policy

As of January 2017, the rent sum policy replaced the income-dependent rent increase policy. With this new policy, the yearly total sum of rent increases is maximized for each individual housing association. The total rent sum per housing association is bound to an increase of inflation + 1%. Rents of individual units may be increased with inflation + 2.5%. In an earlier draft of the new policy, distinction was made between units of which rent exceeds 80% of the maximum allowed rent and units with rents below this boundary. Rent increases of the former were bound by inflation, while rent increases of the latter were maximized at inflation + 2.5% (Ministry of the Interior 2015). Although in the end this distinction was excluded from the policy, this boundary is still applied in this paper (see Table 2). Municipalities and housing associations can also still locally introduce such distinctions. The rent sum policy, additionally, allows rents of households with an income of €39,874 or more may to be increased with inflation + 4%.^3^ The income-dependent rent increases for households with an income higher than €39,874 will not be added to the rent sum, as long as the extra rental incomes are used for projects that are specified in performance agreements between the municipality and the housing association (Aedes 2016b). The maximum rent increase level of inflation + 2.5% also applies to regulated housing rented out by private landlords. Private landlords, however, do not have to comply to the maximum rent sum of inflation + 1%.

**Table 2:** Maximum allowed rent increases of social housing (*Source*: authors)

Housing stock section	Income (€)	Share of max. allowed rent	Max. allowed rent increase
Rent increase	< 39,874	> 80%^a^	Inflation
Rent increase	< 39,874	< 80%	Inflation + 2.5%
Rent increase	≥ 39,874^b^	Any	Inflation + 4%
Harmonization		≤ 100%	Up to 100% of max. allowed rent
Liberalization		N/A	
Total		≤ 100%	Inflation + 1%

^a^This threshold can be adjusted by local government in cooperation with the local housing associations

^b^Pensioners and families of four or more persons are exempted from the extra income-dependent rent increase. The income-dependent rent increase is not included in the total rent sum if the extra revenue is reinvested based on a strategic agreement with the municipality

On the one hand, the rent sum policy restricts the total rent increase housing associations can implement yearly. On the other hand, the new policy provides more space to differentiate rent increases for different (groups of) households. The policy further decentralizes rent restructuring as municipalities and housing association have more room to maneuver and differentiate rent setting. Rent harmonization, however, will limit the space housing associations have to increase rents of sitting tenants as it is also counts for the rent sum. Even if a housing association maximizes the rent sum, they need to decide what rents to increase at what rate.

In the 2008–2014 period, rents could be increased with rates following the inflation until 2013. In the final year of the period observed, rents could be increased with a higher rate of inflation + 1.5% and additional rent increases could be charged for higher earning households. Compared to the period until 2013, the rent sum policy thus allows for a larger yearly rent increase of inflation + 1%. In the 2008–2014 period, however, there were no restrictions regarding rent harmonization. Under the rent sum policy such rent hikes are included in the rent sum. The rent sum policy thus connects the space for harmonizing rents to other rent increases. At the same time, the policy provides more space for rent restructuring through yearly rent increases for sitting tenants. The strategy applied by the housing association will determine the extent to which rents of sitting tenants can be increased and to what extent rents can be harmonized at moments of tenant turnover. The strategy chosen, therefore, is expected to especially influence the gap between new and long-term tenants.

## Forecasting distributive justice outcomes: data and methods

To assess the potential distributive effects of the rent sum policy, the developing affordability for different households is forecasted and valued based on two standards of distributive justice for the 2008–2014 period. In this section, the forecasting method applied will be described (4.1), before discussing the concept of affordability used (4.2) and operationalizing two distinct standards of distributive justice in regards of the distribution of housing affordability (4.3).

### Forecasting

Forecasting will be used for the exploration of short- to medium-term developments of the rent sum policy. The three strategies are forecasted within an institutional setting that is considered as given (van Asselt et al. 2010). This form of research can provide feedback on different possible strategies for the future and thus may help to prevent the use of sub-optimal strategies (Dammers 2010). It differs from scenario-analysis in the sense that uncertainties are placed less central in the different possible futures that are explored (Peterson et al. 2003). The use of multiple forecasts can help to understand the system and the processes of change (Hopkins and Zapata 2007). Although we consider the institutional settings as given, they are, of course, not fixed. By forecasting the effects for the years 2008–2014 that are behind us, the outcomes can be compared with the observed outcomes under the old rent increase policy and practice.

The development of affordability over three two-year periods will be calculated for the new rent sum policy and the former observed situation. For the rent sum policy, three different possible rent increase strategies are forecasted. The rent adjustments and affordability will be forecasted for the 2008–2010, 2010–2012 and 2012–2014 periods. The 2008–2010 period, for example, refers to the period starting on January 1st in 2008 until January 1st in 2010. This includes two moments of rent increase: one in 2008 and one in 2009.

In the forecasts, the income-dependent rent increases are carried out first, since these rent increases are excluded from the rent sum. As mentioned, housing associations can exclude these rent increases from the total rent sum if the benefits are reinvested as agreed with the municipality. The household income limit for income-dependent rent increase of €39,874 has been retroactively indexed using inflation for the previous years. Then, the rent sum is established based on the maximum allowed total rent increase of inflation + 1% (see Table 2). After that, rents in each strategy are increased up to the maximum allowed total rent sum. The increase or decrease in housing allowance for each eligible household is estimated based on the parameters set by the government (Belastingdienst 2015).

A limitation of the applied forecasting method is that potential behavioral responses to the rent increase strategies are not included. Rent increases may impact the relative affordability of a dwelling compared to other housing options (e.g., owner-occupied housing) and consecutive rent increases could result in some households deciding to move-out. The impact of changing behavior over time to our results will be limited since we study a relatively short period of time. Moreover, the period 2008–2014 is analyzed in three two-year sections, within which changing behavior is even more unlikely. We do not further analyze the potentially changing relative price of renting to owning as comparing owning and renting property is notoriously difficult (Conijn et al. 2016). A limitation of cutting up the analysis in 2-year periods is that the cumulative effects of rent increases over a longer period of time become a bit less clear. We will reflect on expected cumulative outcomes of different rent increase strategies.

Housing unit microdata for all social housing units in Amsterdam, including the actual monthly rent charged, is supplied by the Federation of Amsterdam Social Housing Associations (AFWC) and Platform Woningcorporaties Noordvleugel Randstad (PWNR) and is connected to household microdata from Statistics Netherlands (CBS) and the Centre for Policy Related Statistics (CvB), creating a unique dataset. This dataset includes biyearly data for the period 2008–2014, and observations are made at the 1st of January of all even years. Households are included that have an annual income above €8000 (for 2012), a monthly rent of at least €100 (for 2012) and are living in private independent units. This way, outliers, dorms and most students without or with a very low income are filtered out. Students are filtered out as they often do not have to state their income for taxes and as a result would have a very low affordability score, distorting the analysis.

### Affordability

Affordability of housing can be measured in several ways, but the different measures always describe the relation between a household’s income and housing-related expenses (Hulchanski 1995; Stone 2006). A measure of relative residual income determines a household’s spare income after paying housing-related and other necessary expenses. Such a measure takes into account the size and composition of households. In case the measure unveils a lack of affordability, this does not necessarily have to be caused by disproportionate housing expenses (De Groot et al. 2016). It could also, for example, be the result of a very low income or high energy costs. ‘Operationalizing a residual income standard involves using a conservative, socially defined minimum standard of adequacy for non-housing items’ (Stone 1993, p. xx). For such a minimum standard of adequacy for non-housing expenses, minimum budgets for different types of households of the Dutch budget research institute Nibud are used (Nibud 2012). Based on the household size, the building period and size of the dwelling also gas, electricity and water expenses are estimated. Other necessary household expenses are taken from the minimum household budgets of the Dutch budget research institute Nibud, taking into account household size and composition. Using this information, the relative residual income for each household is calculated before analyzing differences in affordability over time and space.

1. residue = disposable household income − (housing expenses + other necessary expenses)
2. residual income (%) = (residue/disposable household income) * 100

### Distributive justice standards

Social housing in the Netherlands is aimed to provide adequate housing for households that ‘because of their income or because of other circumstances have difficulty finding appropriate housing’ (Housing Act, art. 46.1). Housing can be perceived as a ‘primary (spatial) good’ (Basta 2016), a ‘satisfier’ of the universal goal of avoiding serious harm (Doyal and Gough 1991), or the protector of a negative freedom (Merrett 2004). Affordability is one of the key dimensions of housing (Yung and Lee 2012), since adequate housing that is not affordable would mean the households cannot fulfill other necessary expenses. Rent setting policy and strategies, in particular, shape the distribution of rents and affordability of different households. To analyze the impact of rent policy and strategies on households, a distributive justice approach—usually concerning ‘who gets what’—can be applied. To value a certain distribution in terms of distributive justice requires the use of a standard (Cohen 1987). Such a standard can, for example, be maximizing total utility, equal provision for all, or the provision based on the greatest need. Regarding the distribution of affordable housing in Amsterdam, two different standards are used to value distributions of affordability: sufficiency and priority.

Several limitations regarding distributive approaches to justice are noted and voiced by various scholars. These include (see *self*-*identifying reference* for a more elaborate discussion), for example, distributive justice’s neglect to underlying class-structures (MacPherson 1978; Simpson 1980), its tendency to overlook structural deficits of the receipts (Fraser 1995), and its disregard for other important dimensions such as the procedural side of justice, including democratic values (Fainstein 2010), diversity (Fainstein 2010; Young 1990) and recognition (Fraser 1995). Besides, the focus on affordability provides a narrow view on only one of several dimensions of housing. Excluded are, for example, the size of the unit, the accessibility of services, jobs and people, and the quality of the house and its environment. Despite these limitations, the approach is expected to provide a detailed evaluation of the impact of a policy and different possibilities for policy implementation on a central dimension of a basic good for households. Furthermore, the use of two different standards to be discussed provides additional information on the impact of applying different normative standards for policy evaluation.

Frankfurt (1987) argued that the normative concern is not whether people have the same (i.e., equality), but whether people have enough. This author claimed the concern should be the lifting of as many as possible over the threshold of ‘enough,’ instead of aiming for equal provision for all. Of lesser concern in this regard is for those worst off to benefit and see their position improved. Providing to households better off may result in more households reaching a level of sufficiency than by only targeting those worst off. Following this line of argumentation, a distribution should be assessed on whether the individuals or households that are supplied with the good are provided an adequate amount of that good. In terms of housing, adequacy could just be a roof over one’s head in order to protect individuals from physical harm. In this paper, however, the adequacy of a dwelling is assessed in terms of affordability to see whether households are living affordably and what effects distributions of rent increases have on the outcome.

In the case of housing, alternatively, improving conditions of the worst off may well be favored despite lifting a smaller number of households over a threshold. Unlike as in Frankfurt’s (1987) example on the distribution of enough medicine to treat five out of ten patients, not meeting the threshold of affordability may not result in such acute and insurmountable consequences because there is no single natural threshold. The priority standard implies—similar to what has been proposed by Crisp (2003)—that for all households below the threshold priority is given to improve the situation of those worst off (i.e., living least affordably). Next to assessing the affordability for various groups of households, their access to different parts of the city will be observed. Following Frankfurt’s line of thought, one may argue that an adequate dwelling, no matter where in the city, can be considered sufficient. But, as put forward by Casal (2007), the chance to reach a certain level of a good may also be considered. Different parts of the city relate to different levels of locational (dis)advantage and accessibility (Fincher and Iveson 2008) and therefore provide different levels of opportunity for people.

A relative residual income score of zero is used as the threshold for the occurrence of sufficiency. A positive score on this measure means the monthly disposable income of a household is high enough to pay housing-related costs and other necessary costs according to minimum budgets. Since in terms of the sufficiency standard it does not really matter which of the households are lifted passed the threshold—as long as the occurrence of sufficiency is maximized—the occurrence of sufficiency is only regarded for the whole group of tenants. In regard to the second standard applied, priority should be given to households with a significant need. Within this group, the position of those worst off should be prioritized. Those worst off are the groups with the lowest average affordability scores. Consideration will be given to different household types, neighborhoods and groups based on length of residence.

## Socio-spatial distribution of affordability

The outcomes of the rent increase policy and practice in effect in the 2008–2014 period is being compared with the outcomes of the rent sum policy forecasted for the same period. Also, the impact of different ways of policy implementation is explored by forecasting distinct strategies that housing associations could apply. Based on the earlier distinguished sufficiency and priority standards of distributive justice, the effects of both policies and the three different forecasted strategies will be explored. Before discussing results, the three strategies are briefly described.

### Three strategies

The rent sum policy, in comparison with the rent increase policy and practice in the 2008–2014 period that was largely inflation-following, provides more space to apply different rent increase strategies. To compare the two rent increase policy that was in place between 2008 and 2014 and the new rent sum policy, the outcomes of three distinct rent increase strategies housing association could apply given the regulatory boundaries and the limited knowledge they have about their renters (see Table 3). In the *Balanced* strategy (S1), rents of households with a rent above 80% of maximum allowed rent will be increased by inflation. Thereafter, the remaining rent sum will be divided over the other households. The *Harmonization* strategy (S2) will start with harmonizing rent up to maximum allowed for new tenants after tenant turnover within the two-year period. Then, the rents of households with a relatively low rent compared to the maximum allowed rent will be increased, ending with increasing rents of households with relatively high rents. The *Current Tenants* strategy (S3) also prioritizes existing tenants over harmonization, but does not distinguish based on the length of tenancy. These three distinct strategies are selected to show and explore the potential of the rental sum policy.

**Table 3:** Three housing association rent increase strategies. (*Source*: authors)

Rent category	Rent increase strategy		
	S1 Balanced	S2 Harmonization	S3 Current Tenants
> 80% of max. allowed	(1)^a^ Inflation	(3) Up to inflation	(2) Inflation
< 80% of max. allowed	(2) Balanced with harmonization	(2) Up to inflation + 2.5%	(1) Inflation + 2.5%
Harmonization	(2) Balanced with < 80% of max. allowed	(1) Max. possible	(3) Max. allowed
Total^b^	Inflation + 1%	Inflation + 1%	Inflation + 1%

^a^Number in brackets refers to the priority. Rent increase for (1) is firstly established

^b^In all strategies rents for households with an income over €39,874 are increased by inflation + 4% and are excluded from the total rent sum

### Occurrence of sufficiency

The share of households meeting the threshold decreased from about 67.5% at the end of the 2008–2010 period to just over 62% at the end of the 2012–2014 period. Different strategies perform slightly better in different observed periods (Table 4), but there is not one strategy that clearly results in better results of lifting households passed the affordability threshold. The greatest difference between strategies in one period is only 0.2 percentage points. Therefore, it cannot be concluded whether a specific strategy would provide better results when it comes to maximizing the occurrence of sufficiency.

**Table 4:** Share of households with a positive relative residual income for three forecasted strategies (S1, S2 and S3) and actual observations (Obs.). (*Source data*: AFWC, 2008–2014 & CvB/CBS, 2008–2014, calculation by authors)

Period	S1	S2	S3	Obs.^a^
2012–2014	62.4	62.3	62.2	61.0
2010–2012	63.1	63.3	63.1	63.4
2008–2010	67.6	67.4	67.6	67.3

^a^Obs. stands for observed values at the end of the two-year period

In comparison with the observed outcomes under the old policy, the different forecasted outcomes of the rent sum policy all depict lower shares of households with a negative relative residual income than the observed scores for the 2008–2010 and 2012–2014 periods (Table 4), but slightly higher shares for the period in between. The occurrence of sufficiency thus improves in two out of three periods and the policy primarily seems to restrict average rent increases and has an especially positive effect on the average affordability in the 2012–2014 period in which the average rent increase was also higher than inflation.

### Occurrence of priority

To see whether specific types of households may be served well with the proposed rent increase strategies, we regard the average relative residual income for ten different types of households (Table 5). The two household types over the three time-periods with the lowest relative residual income scores are *7. Couple with kids, youngest 12* − and *9*. *Single parent, youngest 12* +. When it comes to the situation of these two household groups, S2 results in the lowest average scores for both household types over all three time-periods (compared to the other strategies). The other two strategies resulted in similar average scores, but the average affordability is slightly higher applying S1 than S3. While S1 and S3 result in higher average scores for the two household types that are worst off in terms of average affordability, these strategies show a more positive outcome for the single parents group (9) than for couples with kids group (7).

**Table 5:** Average relative residual income for different household types in three forecasted strategies (S1, S2 and S3) and actual observations (Obs.). (*Source data*: AFWC, 2008–2014 & CvB/CBS, 2008–2014, calculation by authors)

Household type	S1	S2	S3	Obs.
2012–2014				
1. 1 person, 30 −	22.0	20.3	22.2	18.6
2. 1 person, 30 to 65	8.3	8.0	8.2	7.3
3. 1 person, 65 +	9.2	9.2	9.0	8.3
4. 2 + persons (no kids), all 30 −	14.7	13.0	14.7	12.4
5. 2 + persons (no kids), not all 30 − or 65 +	13.6	13.1	13.4	13.4
6. 2 + persons (no kids), all 65 +	7.0	6.9	6.9	6.8
7. Couple with kids, youngest 12 −	3.6	2.8	3.4	3.4
8. Couple with kids, youngest 12 +	13.3	13.1	13.2	13.4
9. Single parent, youngest 12 −	3.2	1.6	3.0	1.9
10. Single parent, youngest 12 +	8.6	8.0	8.4	7.9
Total	9.3	8.9	9.1	8.6
2010–2012				
1. 1 person, 30 −	20.8	19.8	21.0	19.7
2. 1 person, 30 to 65	10.1	10.3	10.1	10.5
3. 1 person, 65 +	9.8	10.5	9.7	10.2
4. 2 + persons (no kids), all 30 −	16.2	15.5	16.3	15.8
5. 2 + persons (no kids), not all 30 − or 65 +	13.8	13.8	13.8	14.5
6. 2 + persons (no kids), all 65 +	7.8	8.2	7.7	8.3
7. Couple with kids, youngest 12 −	1.5	1.4	1.5	2.0
8. Couple with kids, youngest 12 +	11.2	11.3	11.2	12.0
9. Single parent, youngest 12 −	2.8	2.6	2.9	2.8
10. Single parent, youngest 12 +	10.2	10.4	10.2	10.7
Total	9.7	9.8	9.7	10.2
2008–2010				
1. 1 person, 30 −	22.5	22.0	22.6	21.1
2. 1 person, 30 to 65	13.3	13.2	13.3	13.2
3. 1 person, 65 +	11.8	11.6	11.7	11.7
4. 2 + persons (no kids), all 30 −	18.6	18.2	18.7	18.0
5. 2 + persons (no kids), not all 30 − or 65 +	17.8	17.7	17.8	18.4
6. 2 + persons (no kids), all 65 +	9.9	9.7	9.8	10.0
7. Couple with kids, youngest 12 −	7.6	7.3	7.7	7.9
8. Couple with kids, youngest 12 +	18.6	18.5	18.6	19.3
9. Single parent, youngest 12 −	6.5	5.9	6.6	6.2
10. Single parent, youngest 12 +	13.0	13.0	12.9	13.3
Total	13.3	13.1	13.3	13.4

In comparison with the average relative residual income observed, also S1 and S3 do not in all cases lead to a better result. These two strategies result in a better affordability score in the 2012–2014 period, but show mixed results for the other two periods.

**Fig. 1:**
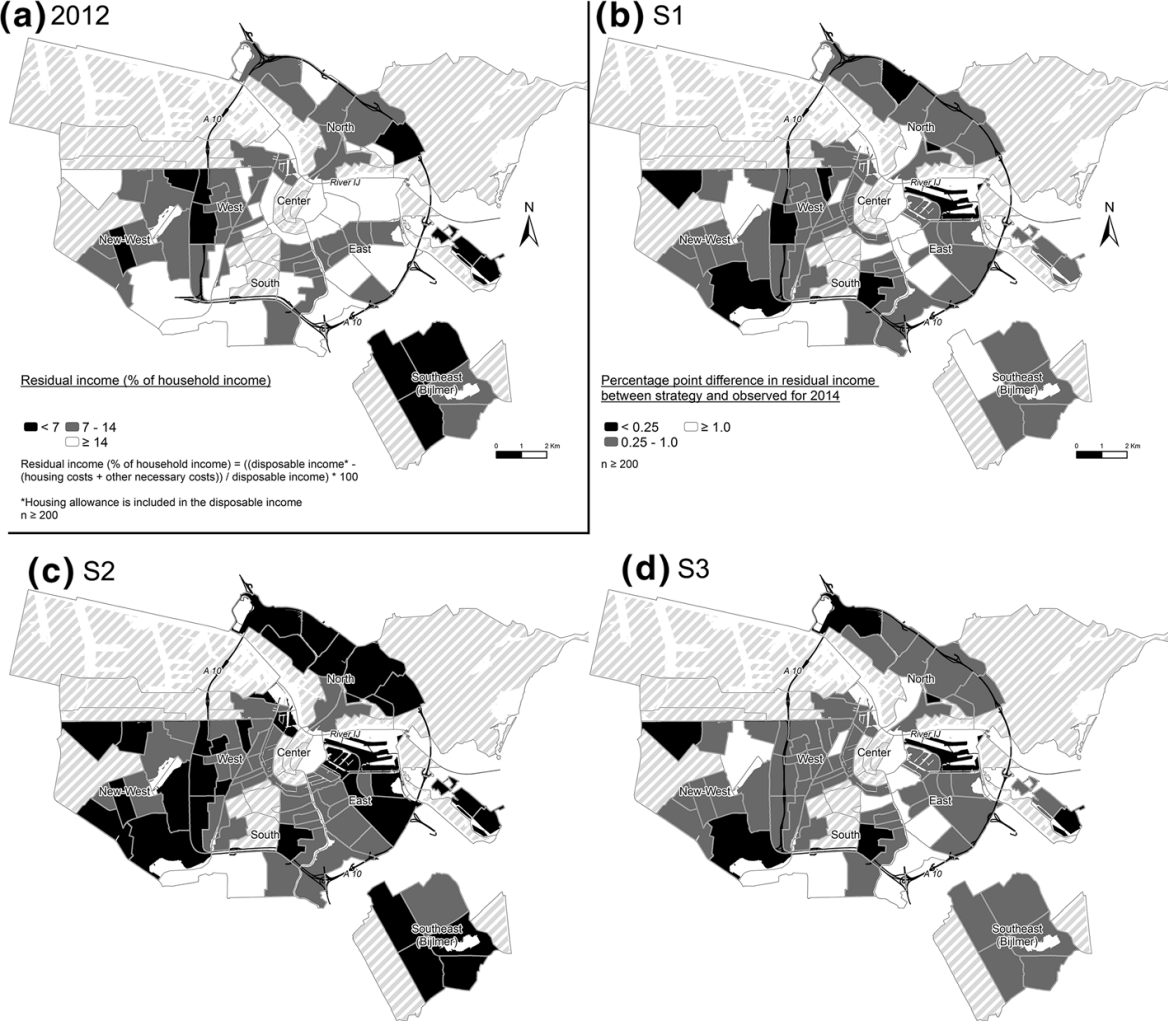
Residual income per neighborhood observed in 2012 (**a**) and per neighborhood difference between observed residual income and residual income based on three forecasted strategies (**b**, **c**, **d**) *Source*: AFWC, 2012–2014; CBS/CvB, 2012–2014; sources layer files: CBS, 2008; CBS, 2009; Ministry of Transport, Public Works and Water Management; made by authors

Figure 1a depicts the average affordability scores in Amsterdam neighborhoods. Areas with multiple neighborhoods with relatively low affordability scores are located in the Southeast, West and East. The area in the East consists of a new large-scale landfill project with units that were recently rented. In the Southeast, tenant turnover is relatively high, resulting in relatively high average rents, since rents can be harmonized if rented out to new tenants. In between 2012 and 2014, S3 (Fig. 1d) provides the highest increases in affordability in these neighborhoods with the lowest average residual incomes in 2012 (Fig. 1a). In S3, all neighborhoods that correspond to the dark-colored neighborhoods provide on average an affordability improvement between 0.25 and 1. For S1 and S2, some of those neighborhoods improve less, though the residual income for one dark-colored neighborhood in Southeast improves by over 1 percentage point in S1. In between 2012 and 2014, S3 seems to provide the best results for the neighborhoods with the lowest average residual incomes. Nonetheless, the results confirm the earlier results that affordability, and thereby distributive justice, is not easily improved by housing associations using generic rent strategies.

Earlier research (self-identifying reference, forthcoming) has also shown a great divide between new and old tenants. Rent increases for sitting tenants have been outpaced by rents for new tenants, resulting in an increasing gap between new and sitting tenants and a premium for *not* moving. One of the aims of the new rent sum policy is to make rents better reflect the quality of dwellings. Table 6 depicts the average residual incomes for different durations of residence. Especially during the first two periods, the average affordability for longer-term tenants slightly decreases, while those of short-term tenants (< 1 and 1–2 years) increase. This trend is more pronounced for S1 and S3 than it is for S2, as expected, since S2 is based on maximizing harmonization of rents (increasing rents of existing stock to new tenants).

**Table 6:** Average relative residual income for different lengths of tenancy in three forecasted strategies (S1, S2 and S3) and actual observations (Obs.). (*Source data*: AFWC, 2008–2014 & CvB/CBS, 2008–2014, calculation by authors)

Years of residence	S1	S2	S3	Obs.
2012–2014				
< 1 year	5.7	1.6	5.4	0.4
1–2 years	6.7	4.3	6.5	2.7
2–8 years	5.2	4.4	5.1	4.8
≥ 8 years	12.2	12.5	12.0	12.1
Total	9.3	8.9	9.1	8.6
2010–2012				
< 1 year	7.2	6.7	7.2	4.1
1–2 years	6.4	6.1	6.5	4.0
2–8 years	5.3	4.8	5.5	6.1
≥ 8 years	12.3	12.8	12.2	13.1
Total	9.7	9.8	9.7	10.2
2008–2010				
< 1 year	8.7	8.3	8.7	6.1
1–2 years	10.6	10.4	10.7	8.7
2–8 years	11.1	10.2	11.2	11.4
≥ 8 years	15.7	15.8	15.6	16.2
Total	13.3	13.1	13.3	13.4

For all periods and all three strategies, the gap between short (1–2 years)-and longer-term households decreases. The increases of affordability for new tenants are significant, and it seems the large gap between new and long-term tenants could be closed for a significant part. In this regards, the new rent sum policy may be expected to have the most significant effect.

The closing of this gap between short- and long-term tenants will mean *not* moving which will provide less of a bonus for tenants. Especially in the longer run, this could increase tenant turnover within the sector and possibly also increase movement into other sectors. Unlike the affordability of those least well-off or the ability of lifting households over a threshold, the equity between short- and long-term tenants, expressed as the average disposable income, increases. This may have positive effects on the functioning of the sector in the long run (CPB/PBL 2016).

## Discussion and conclusion

Next to comparing the new policy with the former policy and practice, three strategies of implementing the rent sum policy have been assessed and compared for their distributive justice effects for different households and neighborhoods in Amsterdam for the 2008–2014 period by combining an ex ante and an ex post evaluation. The new rent sum policy differs from the former policy as it further decentralizes decisions on rent increases. Within boundaries, housing associations can apply distinct strategies to distribute rent increases in different ways. The effects of distributive justice are assessed by the application of two different standards to value the observed and forecasted distributions.

The results show the new policy can only have modest positive effects on both the occurrence of sufficiency and the improvement of affordability for the least well-off (i.e., occurrence of ‘priority’). Comparing the different strategies housing associations could implement, differences are also small. The *Balanced* (S1) and *Current Tenants* (S3) strategies lead to slightly better results in light of the priority standard. These strategies lead to a higher average affordability score for the worst off household groups of single parents with young children and couples with young children. S3 provides the best results looking at the neighborhoods with the lowest average affordability scores. S1 and S3 also provide the best results in terms of decreasing the affordability gap between new and longer-term tenants. This was expected, since the *Harmonization* (S2) strategy prioritizes rent increases for new tenants.

The results confirm earlier findings by De Groot et al. (2016) that rent prices in this context are too crude an instrument to successfully improve the financial situation of renters in the social sector. Moreover, one should look into the more fundamental issue whether the problematic financial situation of households is the result of high rents or low household incomes. In the case of insufficient income, redistributive rent measures will not suffice. The forecasts do show, however, that the observed gap in terms of average affordability between short- and longer-term tenants can be reduced within the rent sum policy, thus contributing to the decline of a well-documented inequity in the Dutch rental sector. This may also have a spatial impact, reducing the gap between high-demand areas with low turnover and low-demand areas with high turnover and therefore also relatively high rents. The fact that the distinction between rents above and below 80% of the maximum allowed rent has been left out of the final draft of the rent sum policy means there is a little more space for housing associations to maneuver. However, movement may also occur in directions other than those anticipated by policy makers. Most significant in this regard is that rents of households living highly affordably but who are charged more than 80% of the maximum allowed rent can be further increased by up to inflation + 2.5% annually. This might over time incentivize some of these households to move into owner-occupation.

Combining the ex post assessment of the distributive effects of rent increases for the 2008–2014 period and the ex ante assessment of such effects of the new rent sum policy by forecasting rent increases for the same period provided a method to compare the old and the new policy landscape under similar conditions. The analysis, however, only accounted for a short period of time. The middle- and long-term effects will strongly depend on how tenants respond to a changing distribution of rents, but also on how social housing rents relate to private rental and owner-occupied housing. A likely outcome is, as reported by for example CPB/PBL (2016), an increase in tenant turnover if the policy leads to a smaller divide between rents of new and long-term tenants (lowering the bonus for not moving) and if rents in the social sector would overall better reflect the quality of the units (smaller gaps between market rates and social housing rents in parts of the city).

The strategies applied for the forecasting exercise have been based on the limited information housing associations have, which restricts their implementation options. For example, they know the income level, and household size and composition at point of entry, but do not if and when those factors change. To be able to enact the income-dependent rent increase, landlords are only provided with an indication by the Dutch tax services of the income category a household belongs to. Under the new policy, landlords will only know whether a household earns more or less than €39,874, which does not enable further stratification. The size and composition of the household—though crucial when it comes to affordability—is not included in any way. Thus, the question can be raised whether the distribution of affordability through rent increases could be more just if more information would be provided as input. The effects, however, should be weighed against the consequences it would have on household privacy and tenant security.

While the results show modest potential effects of the rent sum policy on the distributive justice, in practice it has already been further complicated by the introduction of another policy. The new policy on appropriate allocation (*passend toewijzen*) prescribes housing associations to allocate 95% of lettings each year to households entitled to receive housing allowance (i.e., single-person households with an income below €22.100 and multi-person households with an income below €30.000) to a dwelling with a rent level below the housing allowance rent limit (€587 for one- and two-person households and €629 for larger households in 2016) which is lower than the social housing limit of €710. This strongly restricts possibilities for harmonization. At the same time, harmonization will be housing associations primary concern. Determining how much of the total rent sum increase will be left for regular rent increases may also be more difficult to determine. Despite these additional limitations, it will still be possible to make decisions on how to distribute rents among current tenants, taking into account the impact on affordability of different households.

Incorporated in the forecasting exercise is the partial compensation many tenants receive for increasing rents by corresponding changes in the housing allowance received. Because rent increases are only partially compensated, lower-income households are still affected by increasing rents.^4^ More market-oriented rents, without changing the housing allowance scheme, would therefore also negatively affect already worst off households. While the rent sum policy aims to make rents better reflect housing quality, the increased freedom to set rent increases also provides more space to choose alternative paths. Similar to England and Belgium rent setting incorporates different aims. Unlike the Dutch case, rents are determined centrally in England and Belgium. The decentralization of rent increase decisions in the Netherlands is not expected to lead to major distributive justice improvements, but it enables the ability to reckon with local needs. The results furthermore show the policy change may also result in a less ‘flat’ rent distribution in which the housing quality plays a slightly larger role in rent setting. Not knowing the need of households in terms of affordability in combination with the partial housing allowance compensation, however, greatly limits opportunities to make rents better and more consistently reflect the quality and value of housing and complicates the search for positive effects on the justice of affordability distributions.

## Footnotes

1 Both housing associations and private landlords rent out regulated and unregulated dwellings. Private owners make up 26% of the total rental stock in the Netherlands, while 88% of the rental stock is rent controlled. A large share of institutional and privately owned rental housing is rent controlled.

2 As a result of recent amendments to the valuation system for newly rented-out units, as of 2017 a larger share of the maximum allowed rent is now determined by the vacant possession value of the dwelling. Some other components—e.g., scarcity points, dwelling type, nuisance causing situations around the house—are now left out of the calculation (Ministry of the Interior 2015).

3 In the final version of the policy, households existing of members above the retirement age and households consisting of four or more persons.

4 A household multi-person household with an annual income of €16.000 and a rent of €500, for example, would in the year 2014 see €5,25 of a €15,00 (equal to 3%) rent increase compensated through housing allowance. This would result in a 0.73 percentage point decrease of the relative residual income.
